# Are They the Same for All People? Nurses’ Knowledge about the Basic Human Needs of People with Disabilities

**DOI:** 10.3390/bs13010068

**Published:** 2023-01-12

**Authors:** Marija Ljubičić, Ivona Burčul, Ivana Gusar, Sonja Šare

**Affiliations:** 1 Department of Health Studies, University of Zadar, Splitska 1, 23000 Zadar, Croatia; 2 Rehabilitation Centre Philip and Jacob, Put Primorja 56, 23000 Zadar, Croatia; 3 Medical School Ante Kuzmanića Zadar, Franje Tuđmana 24G, 23000 Zadar, Croatia

**Keywords:** nurse, knowledge, basic human needs, Maslow’s hierarchy, people with disabilities, questionnaire, validation

## Abstract

Nursing care involves a continuous interaction between nurses and people with disabilities. This has created a need for assessment tools that measure nurses’ knowledge about the basic human needs of people with disabilities. The aim of this cross-sectional study was to develop a Knowledge of Basic Human Needs Scale and investigate nurses’ levels of knowledge about the basic human needs of people with disabilities and their association with nurses’ education. Data were analyzed using principal component analysis to test the construct validity and to identify factors using principal varimax rotation. The reliability estimate was based on Cronbach’s alpha coefficient. Linear regression models were used to assess the association between knowledge about basic human needs and predictors. Factor analysis extracted eight factors, explaining 66.3% of the total variance. The sampling adequacy, criterion validity, and internal consistency were satisfactory. The nurses’ levels of education was associated with their knowledge about the basic human needs of people with disabilities. The questionnaire constitutes a valuable contribution to improving nurses’ knowledge and practice, as well as the quality of healthcare, and it provides a contribution to improving the quality of life for people with disabilities.

## 1. Introduction

Worldwide, more than one billion people live with a disability [1]. The United Nations defines people with disabilities as “those who have long-term physical, mental, intellectual, or sensory impairments, which, in interaction with various barriers, may hinder their full and effective participation in society on an equal basis with others” [2]. In this definition, a crucial segment constitutes barriers, which often originate from a person’s environment. The World Health Organization (WHO) emphasizes that people with disabilities face barriers in accessing health services and have worse health outcomes than people without disabilities [1]. Moreover, when people with disabilities come to health institutions, they are at risk of receiving inadequate healthcare as a result of many different factors [1,3,4]. This depends not only on the disability of a person, but also on the potentially inadequate health services that health workers—especially nurses—can provide [5]. Nurses are the first healthcare workers that people with disabilities meet upon arriving at health institutions. Hence, nurses need to understand the basic human needs (BHNs) of people with disabilities—not just their clinical conditions [3]. Nurses’ services should be organized to ensure the highest level of healthcare and improve the quality of life for people with disabilities [6,7,8].

Abraham Maslow proposed a classification of basic needs into five categories: physiological, safety, belongingness, esteem, and self-actualization. He hypothesized that these needs are essential for individual psychological health, but that the physiological and safety needs are more powerful than the higher needs [9]. Moreover, affiliative needs are in the middle of this hierarchical structure, highlighting their strong association with other BHNs [9]. The BHNs of people with disabilities are the same as the needs of all people—to be independent, to love, to be loved, and to be successful are only some of the human needs of all people, including people with disabilities; the only difference is in the ways and possibilities through which to achieve them. For example, all people want to eat and drink adequately, move from one place to another, maintain desirable postures, dress and undress, keep their body clean, maintain their body temperature within a normal range, sleep and rest, avoid dangers, communicate with others by expressing emotions, needs, fears, or opinions, learn, and satisfy their curiosity [10]. People with disabilities have the same human needs, but they sometimes need support to achieve these aims [11]. 

Nurses need to be aware that health cannot be achieved without satisfying the BHNs of people with disabilities, following Maslow’s hierarchy. The nursing theorists Virginia Henderson and Dorothy Orem wrote that the nurse’s role is to help patients to satisfy their needs, maximize their self-care skills, and achieve independence [10]. Nurses help people with disabilities regardless of their age, disability or other medical condition, type of care setting, and/or cultural background [12]. Studies have provided evidence that human needs are fundamental in every patient–nurse relationship [12]. In order for nurses to determine which need is dominant at a certain moment, it is necessary to perform an assessment. Nursing assessments take place constantly and in continuous interaction between the nurse and the disabled person. Since BHNs are the same for all people, but their intensity and the ways in which they are met are different, the assessment should be performed in a way that takes a holistic approach. To be able to do this, the nurse needs to have knowledge about the hierarchy of BHNs, as well as the psychological, social, and spiritual circumstances of their occurrence.

The importance of including disability issues in the curricula of schools of medicine/nursing and health institutions, and of designing model disability curricula for healthcare workers, is part of the WHO’s global disability action plan [1]. Nevertheless, the explicit content that focuses on disability as a multidimensional construct is insufficient [8,13]. Inadequate education of nurses may result in inappropriate nursing care, while people with disabilities may face considerable barriers to healthcare because of a lack of knowledge among nurses [13]. Given the evidence from many studies of a lack of knowledge and negative attitudes, poor communication on the part of nursing staff, compromised care, and fears related to the quality of care of people with disabilities, there is a need to conduct research to analyze associations between nursing education and knowledge about the BHNs of people with disabilities in order to improve the quality of nursing care for these people [3,14]. Studies show that nursing education specific to people with disabilities may improve the knowledge of BHNs and improve the quality of nursing care [8,13,15]. Increasing knowledge among healthcare professionals—especially nurses—and the provision of tools to screen, diagnose, treat, and provide nursing care for all people with disabilities with dignity are crucial aims to improve the health status of people with disabilities [3,8,16].

To the best of our knowledge, this is the first study to investigate nurses’ knowledge about the BHNs of people with disabilities according to Maslow’s motivation theory and to develop a questionnaire for its assessment. Previously, studies based on this topic did not examine nurses’ knowledge about the needs of people with disabilities [17,18,19]. There has been no research on the knowledge of nurses about these complex nursing tasks, especially with respect to people with disabilities. Therefore, it is important to assess current nurses’ knowledge about the BHNs of people with disabilities so that nurses can receive an adequate education. This is especially important because nurses have a responsibility for people with disabilities and the planning of nursing care for such people. Generally, BHNs—especially in people with disabilities—are often neglected and poorly understood. From the perspective of people with disabilities, nurses need to improve their handling of communication, competence, attitudes, and patient safety—especially during hospitalization—constituting one of the crucial reasons to develop a scale for the assessment of nurses’ knowledge [14]. For these reasons, all areas that require improvement must be identified, closely studied, and highlighted. This is an especially important research problem not only for the nursing profession, but also for other important areas of the so-called helping professions, such as physicians, rehabilitators, physiotherapists, psychologists, and speech therapists. 

We aimed to develop and test an instrument to measure nurses’ knowledge of the BHNs of people with disabilities using Maslow’s hierarchy of needs. Additionally, this study aimed to investigate the association of nurses’ levels of knowledge about the BHNs of people with disabilities and the categories of Maslow’s hierarchy of needs with nurses’ sociodemographic characteristics. We hypothesized that the nurses’ knowledge about the BHNs of people with disabilities would be associated with their education, length of service, and age, and that nurses with higher levels of education and longer service would have better knowledge. We assumed that knowledge about the categories of Maslow’s hierarchy of needs would be interconnected and provide answers for possible improvements in healthcare. Additionally, we assumed that this questionnaire could be an important instrument for the assessment of nurses’ knowledge to detect crucial segments of nursing care that require improvement in the knowledge and skills of nurses, as well as of other professionals involved in healthcare for people with disabilities. If these assumptions are accurate, it will confirm the importance of education in the area of healthcare for people with disabilities, which is not well represented. The learning outcomes of this segment of education for all healthcare professionals should necessarily include the acquisition of competencies necessary for working with people with disabilities and children with developmental disabilities. This may have a positive impact on improving healthcare for people and children with disabilities, as well as their inclusion in society.

## 2. Materials and Methods

### 2.1. Sample and Procedure

A cross-sectional survey was administered to a convenience sample of 160 nurses. The nurses were recruited from General Hospital Zadar. The study took place from June to September 2018. At the time of the research, 550 nurses were employed in the hospital. The exclusion criteria were nurses on long sick leave and nurses on maternity leave. On the basis of these criteria, to prevent bias, we excluded 52 nurses. Unemployed nursing students who were in clinical education in the hospital at the time of the study were not included. Accounting for the decline in participation, along with the exclusion criteria, the response rate was 29.1%.

The study was approved by the Human Research Ethics Committee of the Zadar General Hospital (02-6432/18-5/18). All nurses gave their informed consent for inclusion before participating in the study. The research was conducted in accordance with the ethical standards of the Declaration of Helsinki.

### 2.2. Sociodemographic Questionnaire

The sociodemographic data analysis included the assessment of age, gender, level of education, length of service, and studying at university.

### 2.3. Questionnaire

Nurses completed a self-administered, constructed questionnaire titled “Knowledge of Basic Human Needs Scale” (KBHNS) to assess the nurses’ knowledge about the BHNs of people with disabilities.

The questionnaire was constructed by the first author (an expert in this area) and the second author (a nurse with professional experience in caring for people with disabilities). According to the relevant literature, we determined the content of the questions, covering all levels of Maslow’s hierarchy of human needs, and developed the final questionnaire [11,20,21]. Before applying the instruments, five experts reviewed and evaluated the contents of the final versions of the questionnaire. These experts were highly knowledgeable about the field of interest and were potential users of the scale. They included two experts on the theoretical meaning of the construct of BHNs and Maslow’s hierarchy, as well as three nurses with over 10 years of professional experience and graduate nursing. All of the panel experts agreed that the questionnaire’s contents were valid and that the items had highly relevant significance for each domain. These findings confirm that the contents of the questionnaire adequately represent the constructs of knowledge that nurses need to have when providing nursing care for people with disabilities. Before applying the questionnaire, we performed a pilot test on five nurses in order to confirm that the questionnaire was understandable, and that it would provide answers within the expected range.

The KBHNS contains 30 items combined into six components: general knowledge about people with disabilities; physiological needs; safety needs; affiliative needs; self-esteem needs; self-actualization needs. Each component contains five items.

The general knowledge component includes items of knowledge about the differences in the BHNs of people with and without disabilities, the independence and ways of meeting the needs of people with disabilities, and the interaction, equal participation, and inclusion of people with disabilities in society. The knowledge about physiological needs, rest, and mobility of people with disabilities includes knowledge about self-care, rest, sleep, reduced mobility, stimulation, and delays in satisfying physiological needs. The safety needs include knowledge about hygiene, avoiding harmful influences from the environment, decubitus prevention, falls, injury, and infection. The knowledge about affiliative needs includes items about love, belonging, communication, social isolation, and spiritual needs. The fifth group of items includes knowledge about self-esteem needs, self-confidence, self-concept, and strengthening the remaining personal abilities, while the sixth group includes self-actualization, counseling, and possibilities for increasing knowledge about specific problems among people with disabilities.

Answers were defined by a five-point Likert scale: 1 = completely incorrect; 2 = incorrect; 3 = partially correct; 4 = correct; 5 = completely correct. Three items were negatively oriented and were scored in reverse. The sum for each of the components ranged from five to 25. The sum of all 30 items in the whole questionnaire ranged from 30 to 150. Higher scores denoted greater knowledge about BHNs (Supplementary Table S1).

### 2.4. Data Analysis

Statistical analysis was conducted with SPSS Statistics v21.0 (IBM, Armonk, NY, USA). Values of *p* < 0.05 were deemed to be statistically significant. The data distribution was analyzed using the Kolmogorov–Smirnov test. We used the median and interquartile range for descriptive statistics and used complete case analysis. Categorical variables were displayed as absolute numbers and percentages. The Spearman coefficient test was used to analyze the correlation among numerical variables. On the basis of the data collected, we performed the Mann–Whitney U test and the Kruskal–Wallis test to evaluate differences in knowledge about BHNs according to the nurses’ sociodemographic characteristics. The validation of the questionnaire included a factor analysis to test the validity and reliability of the questionnaire. The factor structure of the KBHNS was tested through one of the explained factor combinations and was accepted. The reliability estimate, i.e., internal consistency, was based on Cronbach’s alpha coefficient for the entire questionnaire, as well as for each of the factors given in the factorial analysis. 

Thirty items relating to nurses’ knowledge were analyzed using factorial analysis. We applied principal component analysis (PCA) to the KBHNS questionnaire to test the construct validity and identify factors, using principal varimax rotation component analysis and a cutoff of >0.40 for absolute factor loadings to suppress small coefficients for better interpretability. We used the Kaiser–Meyer–Olkin measure of sampling (≥0.80) to assess the adequacy of the sample size and Bartlett’s test of sphericity (*p* < 0.05) to confirm the adequacy of the data for factor analysis. We used a criterion of eigenvalues ≥1 for factorial extraction, in order to display only the factors that met this criterion. We used the criterion that the minimal number of respondents providing usable data for the factorial analysis should be five times the number of variables being analyzed. Thus, an appropriate sample size based on the 30 items on our questionnaire necessitated a minimum sample of 150 nurses [22].

Several linear regression models were created to assess the associations between the main outcome—knowledge about the categories of Maslow’s hierarchy of human basic needs—and its predictors (i.e., age, gender, education, length of service, and studying at a nursing university), as well as to test associations between domains of nurses’ knowledge and their predictors. To prevent statistical bias, all predictors were entered into the model at the same time. We performed a priori power analysis using G*Power v3.1.9.4 software (Universität Kiel, Kiel, Germany). Based on 13 predictors in multiple linear regressions, with effect size f^2^ = 0.15, power 1 − β = 0.80, and significance level α = 0.05, it yielded a required sample of 131 nurses. We performed a posteriori power analysis based on a sample of 160 nurses and 13 predictors, yielding a satisfactory power (1 − β = 0.89).

## 3. Results

### 3.1. Sociodemographic Data of the Sampled Nurses

The nurses ranged in age from 21 to 62 years. The median age of the sample was 37.0 years (IQR = 17.4). Around one-third of the nurses (34%) were aged between 31 and 40 years. Most of the nurses (94.4%) were female, and 5.6% were male. A total of 61.3% of the nurses had a high-school degree, while 35.6% were educated to bachelor’s level. Only 3.1% of the nurses had a master’s degree. Around 84.4% of the nurses worked in a stationary department, and 15.6% of the nurses worked in the outpatients’ clinic. There was an even distribution of length of service, at ~30% for each of the decades. Around 11% of the nurses had attended university-level nursing education (Table 1).

### 3.2. Reliability and Validity of Questionnaire

The Kaiser–Meyer–Olkin measure of sampling adequacy (N = 160) was KMO = 0.82, while Bartlett’s test of sphericity was significant (χ^2^(435) = 1920.82, *p* < 0.001), indicating that properties of the correlation matrix justified the factor analysis carried out. Furthermore, our KMO measure (0.82) and Bartlett’s test (*p* < 0.01) showed an adequate sample size and moderate intercorrelations, indicating an appropriate statistical analysis for the given data.

The levels of the nurses’ knowledge about the BHNs of people with disabilities were grouped into eight factors explaining 66.3% of the variance. The loadings of all 30 items grouped into these eight domains are shown in Table 2. The first factor, “knowledge about self-care”, explained 11.4% of the variance in the dataset. The second factor, “knowledge about self-actualization”, explained 11.1% of the variance. The third factor, “knowledge about communication and belongingness”, explained 9.6% of the variance. The fourth factor, “knowledge about safety”, explained 9.5% of the variance. The fifth factor, “knowledge about self-esteem”, explained 6.9% of the variance. The sixth factor, “general knowledge and view of advising”, explained 6.5% of the variance. The seventh factor, “knowledge about independence”, explained 6.0% of the variance. The eighth factor, “knowledge about belief”, explained 5.4% of the variance in the dataset. The reliability (Cronbach’s alpha) for the first to eighth factors ranged from α = 0.85 to α = 0.62. Cronbach’s alpha achieved for the entire questionnaire was α = 0.84 (Table 2).

### 3.3. Nurses’ Knowledge about the Basic Human Needs of People with Disabilities

The nurses’ average knowledge about BHNs was Mdn = 4.0 (IQR = 0.5) (Supplementary Table S2). Nurses with master’s degrees showed higher levels of general knowledge (Mdn = 21.0 (IQR = 7.5); *p* = 0.008) and self-actualization knowledge (Mdn = 22.5 (IQR = 3.8); *p* < 0.001) with respect to people with disabilities than the other education groups. Nurses with a longer length of service (*p* = 0.015) and nurses who had not studied at a nursing school (*p* = 0.003) reported higher knowledge about the safety needs of people with disabilities (Supplementary Table S2).

### 3.4. Associations between Knowledge about Basic Human Needs and Sociodemographic Characteristics

The linear regression models confirmed an association between nurses’ knowledge about basic human needs, age, education level, university study, and workplace (Table 3). Nurses’ general knowledge about people with disabilities was relatively strongly associated with older age (β = 0.76; *p* = 0.051), which was also moderately associated with their knowledge about affiliative needs (β = 0.20, *p* = 0.014) and self-actualization needs (β = 0.16, *p* = 0.036). Nurses with only a high-school education had lower knowledge about self-actualization (β = −0.44; *p* = 0.027), while no associations were found between level of education and knowledge about other basic human needs. Studying at a university was associated with knowledge about physiological needs (β = −0.14, *p* = 0.028) and safety needs (β = 0.17, *p* = 0.006). Working at a stationary department was negatively associated with knowledge about safety needs (β = −0.13; *p* = 0.051). Length of service showed a certain trend toward a significant association with general knowledge about people with disabilities (β = −0.65; *p* = 0.094). Knowledge about physiological needs was moderately associated with knowledge about safety needs (β = 0.50, *p* < 0.001), and vice versa (β = 0.54, *p* < 0.001), and both were moderately associated with affiliative needs (physiological needs: β = 0.29, *p* = 0.002; safety needs: β = 0.17, *p* = 0.034). Knowledge about the self-esteem needs of people with disabilities was positively associated with self-actualization (β = 0.41; *p* < 0.001), while knowledge about self-actualization was associated with all components of knowledge about basic human needs, but most strongly with self-esteem (β = 0.45, *p* < 0.001). According to these associations, the linear regression highlighted important predictors for dependent variables. General knowledge and physiological needs were predictors of affiliative needs and self-actualization, while safety needs were predictors of physiological needs, affiliative needs, and self-actualization. Affiliative needs were a predictor of general knowledge, physiological needs, and safety needs. Self-esteem needs were a predictor of self-actualization, and vice versa, while self-actualization was a significant predictor of general knowledge, physiological needs, and safety needs. Age was a strong predictor of general knowledge, while level of education was a predictor of self-actualization, and university study was a predictor of physiological needs and safety needs. The overall significance of each model is shown in Table 3.

### 3.5. Associations between Domains of Knowledge and Sociodemographic Characteristics

The linear regression models confirmed the association between domains of knowledge about BHNs and nurses’ sociodemographic characteristics (Table 4). In comparison with those with a master’s degree, nurses with a bachelor’s degree had lower knowledge about self-esteem (β = −0.44; *p* = 0.045) and belief needs for people with disabilities (β = −0.43; *p* = 0.029), while nurses with a high-school education had lower knowledge about self-actualization (β = −0.43; *p* = 0.017) and self-esteem (β = −0.63; *p* = 0.005). Length of service showed a strong association with the domain of knowledge about safety needs (β = 0.69; *p* = 0.049). Working at a stationary department was negatively associated with the domain of knowledge about independence (β = 0.19; *p* = 0.027). Female gender and studying at a university showed a certain positive trend toward significant association with the belief domain of people with disabilities (β = 0.13, *p* = 0.073; β = 0.13; *p* = 0.064, respectively). Nurses’ knowledge about self-care needs was positively associated with the belongingness domain (β = 0.21; *p* = 0.004) and safety (β = 0.17; *p* = 0.041). Knowledge about self-actualization was positively associated with the domains of general knowledge and view of advising (β = 0.39; *p* < 0.001), knowledge about safety (β = 0.26; *p* = 0.007), and knowledge about needs for belief among people with disabilities (β = 0.19; *p* = 0.048). The domain of knowledge about belongingness was associated with knowledge about self-care (β = 0.29, *p* = 0.004), safety (β = 0.26, *p* = 0.008), and believing (β = 0.35, *p* < 0.001). Nurses’ knowledge about safety was associated with self-care (β = 0.18, *p* = 0.041), self-actualization (β = 0.21, *p* = 0.007), and belongingness (β = 0.20, *p* = 0.008). The domain of general knowledge and view of advising was associated with self-actualization (β = 0.31, *p* < 0.001) and belongingness (β = 0.15, *p* = 0.045). Nurses’ knowledge about belief was associated with the domains of self-actualization (β = 0.15, *p* = 0.048) and belongingness (β = 0.27, *p* < 0.001) needs, while knowledge about self-esteem was negatively associated with the domain of knowledge about self-care (β = −0.24, *p* = 0.002). Considering these results, female gender and length of service were negative and positive predictors for safety, respectively. Education was a predictor for safety, self-actualization, and beliefs. Working at a stationary department was a negative predictor for independence. Self-care was a predictor for belongingness, safety, and self-esteem. Self-actualization and belongingness were predictors for safety, general knowledge and advice, and beliefs. Safety was a predictor for self-care, self-actualization, and belongingness. General knowledge and beliefs were predictors for self-actualization and belongingness, while self-esteem was a predictor only for self-care. The overall significance of each model is shown in Table 4.

## 4. Discussion

Our results indicate that the nurses showed a good level of knowledge of the BHNs—including knowledge of physiological needs, safety needs, affiliative needs, self-esteem needs, and self-actualization needs—of people with disabilities. These knowledge domains were associated with general knowledge of people with disabilities, as well as with the nurses’ sociodemographic characteristics. The results show that higher levels of nurses’ education were associated with higher levels of nurses’ knowledge about the BHNs of people with disabilities. Additionally, a longer length of service was shown to have a positive impact on nurses’ knowledge. These results are consistent the findings of other studies showing that higher levels of nurses’ knowledge are strongly associated with nurses’ education and professional experience [23,24,25]. However, our research showed that general knowledge was associated with knowledge of the needs for belongingness and self-actualization of people with disabilities. Additionally, nurses with a master’s degree education had higher knowledge of the highest-level needs of Maslow’s hierarchy.

The association between higher levels of education and self-actualization may indicate that nurses with higher levels of education have more specific knowledge about these crucial needs for achieving the highest possible level of self-fulfillment for people with disabilities. The absence of an association between the level of education and other knowledge about BHNs—such as physiological, safety, affiliative, and self-esteem needs, as well as general knowledge about people with disabilities and the domains of self-care, safety, independence, belongingness, and advice—may indicate that better education of nurses’ serving as members of multidisciplinary teams is strongly needed. This may indicate that the basic knowledge shown by the nurses in this research should be improved with specific knowledge related to the needs of these people and better activities in nursing curricula. The regression model indicates the connection between general knowledge and affiliative needs and the need for self-actualization. Additionally, the strong connection between age and general knowledge of nurses about people with disabilities indicates that, with increasing age, nurses’ levels of general knowledge increase, and that lifelong and professional experience may also be among the predictors of nursing knowledge [24,26]. It is possible that nurses acquire their knowledge about BHNs informally, i.e., through various lectures, literature, magazines, or other media, or by exchanging knowledge and experience with colleagues and coworkers. These results are confirmed by the findings of other studies that emphasized the need for development of competencies and education of nurses in this field [27]. These competencies may involve the skills for the assessment of disability and health conditions, competency in patient-centered care, understanding of the roles of members of multidisciplinary teams, perception of quality of life, and the legal framework of anti-discrimination legislation [3,16]. 

Increasing the knowledge among healthcare professionals is crucial for improvement of the health status of people with disabilities [16]. However, this may be insufficient to change attitudes, instill the specific knowledge required, or develop specific skills of nurses with regard to the health needs of people with disabilities [3]. In nursing, as in other health professions, experience plays a crucial role in the learning and acquisition of nursing skills. Studies have defined learning as the process of creating knowledge through the transformation of experience [28,29]. In our study, we found a strong association between length of service and factors of knowledge about the safety of people with disabilities. However, we did not find any association between length of service and knowledge of other BHNs or the domain of loading knowledge. Similar findings have also been reported in other studies, where health professionals did not show good knowledge about people with disabilities, despite their experience [30]. In our study, the knowledge of the nurses was satisfactory, but these results may indicate informal means of nursing education that are not specifically directed to the needs of people with disabilities. The possibility of inter-professional education, simulations, clinical experience, and visits to community facilities and organizations to meet people with disabilities are just some of the many different ways of learning about disabilities. These innovative formal methods in curricula have the potential to broaden the professional understanding of disability among nursing students [3]. On the other hand, experience contributes to increasing the levels of knowledge and skills acquired while studying. Informal learning represents an increasingly widespread form of learning and education. Together with formal learning, it represents conscious and organized learning, accompanied by motivation, with the aim of acquiring certain knowledge and developing skills and attitudes, i.e., the acquisition of competencies within the framework of nurses’ personal and professional activities. 

Future research should consider factors that influence nurses’ levels of knowledge. In nursing, this concept implies the active participation of nurses in all forms of education, including formal education (i.e., studies at faculties) and informal education (e.g., acquisition and development of skills needed in the workplace, as well as informal education through the exchange of knowledge and experience among colleagues and coworkers) [28,29]. Moreover, informal education, learning transfer, and clinical career level may influence clinical performance [31]. Studies confirm that approximately 70–90% of learning takes place in the workplace through informal interactions, which could explain the good knowledge of the nurses in our research and its association with the nurses’ length of service [31].

Despite the fact that disability can be extremely diverse, people with disabilities may experience greater vulnerability, treatment without consent, or inadequate communication without an understanding of their needs [1]. According to the WHO’s global disability action plan for better health for all people with disabilities, the emphasis should be on the need to improve access to health services, strengthen and build human resource capacity, and educate healthcare workers—especially nurses. The WHO also emphasizes the need to develop evidence-based tools to assist and model disability surveys and to enable national reporting from health and education ministries and national centers of excellence [1]. Accordingly, this questionnaire may be one of the tools that can be used to access a crucial segment of nurses’ knowledge in order to detect areas of deficit on which it is necessary to act in order to improve nursing practice for people with disabilities.

Despite these strengths, this study had some limitations. First, this was a cross-sectional study; hence, we could not prove causality, but it may help to generate causal hypotheses. Second, we used a convenience sample from one hospital in Croatia, which may have also influenced the results. Third, we had to develop a new instrument. Despite our results being very encouraging, showing good reliability and enough correlations for factor analysis, future research is needed in order to additionally show the reliability and validity of this questionnaire.

Hence, because nurses continuously interact with patients and are responsible for their nursing care and care planning, it is necessary to increase their knowledge. Specifically, studies have shown that people with disabilities tend to have unmet BHNs because of inadequate education, deficits in knowledge and skills, and negative attitudes of nurses and nursing students, with consequent deficits in the quality of healthcare [13,32]. Nurses are advocates for people with disabilities, as well as for patient autonomy and the right to self-determination. If their knowledge about these needs is insufficient, this can present a problem in promoting access to regular and high-quality healthcare [25]. Knowledge can certainly be best conveyed by nurses who are skilled practitioners with experience in this area, as well as by those with a high level of education and research experience, in order to strongly connect the theory and practice of care for people with disabilities. Systematic evaluation is needed to assess how nurses’ knowledge influences practice [8]. Additionally, nurses will need to collaborate with other professionals, adapt to new technologies, and engage in new professional roles related to caring for people with disabilities. These roles require the highest levels of education, especially when providing care for people with disabilities and their families [33]. An especially important aspect of providing complete healthcare is the collaboration of nurses with other disciplines within the healthcare system. Ultimately, this is an important issue because people with disabilities need long-term care owing to their varied needs [34]. For example, a combination of physical, psychological, and cognitive deficiencies can create an impression that it is necessary to first take care of the physical or psychological issues, i.e., the problem that is most apparent and limiting [35,36]. Therefore, it is important to adopt a holistic and multidisciplinary approach to cover all levels of Maslow’s hierarchy of needs with a positive impact on a person’s state [11,35,36]. Comprehensive rehabilitation approaches are associated with good knowledge of the needs of people with disabilities. Each healthcare professional plays a key role in identifying and analyzing the independence, strengths, and needs of people with disabilities. Accordingly, knowledge about the BHNs of people with disabilities among all members of the multidisciplinary team (e.g., nurses, physicians, rehabilitators, physiotherapists, psychologists, and speech therapists) has great importance in the provision of care for these populations. Educational strategies to ensure adequate knowledge among all professionals about the needs of people with disabilities may help in improving the care provided to such people [14].

## 5. Conclusions

This instrument may constitute a very valuable input for improving the knowledge and practice of not only nurses, but also other professionals involved in the provision of healthcare to people with disabilities. Additionally, this study makes an important contribution to the continuous assessment of knowledge in this field to increase the quality of healthcare for this vulnerable population. A higher quality of curricula—including formal and informal education for the working processes of all members of multidisciplinary teams—is urgently needed. The role of nurses and other team members is irreplaceable in recognizing BHNs, achieving patients’ independence, and ensuring the inclusion of people with disabilities in society. Moreover, comprehensive rehabilitation approaches and collaboration within coherent teams and between all professionals involved in healthcare would strongly benefit the inclusion of people with disabilities in society.

## Figures and Tables

**Table 1:** Nurses’ sociodemographic characteristics and levels of knowledge about basic human needs (N = 160).

Age (Years), Mdn (IQR)	37.0 (17.4)
Age groups, N (%)	
21–30	42 (26.9)
31–40	53 (34.0)
41–50	38 (24.4)
51–60	22 (14.1)
61 and over	1 (0.6)
Gender, N (%)	
Male	9 (5.6)
Female	151 (94.4)
Education, N (%)	
High-school degree	98 (61.3)
Bachelor’s degree	57 (35.6)
Master’s degree	5 (3.1)
Length of service (years), Mdn (IQR)	16.5 (17.0)
Length of service; year groups, N (%)	
Fewer than 10	45 (29.0)
11–20	47 (30.3)
21–30	45 (29.0)
31 and over	18 (11.6)
Study at a nursing university, N (%)	
Yes	17 (11.0)
No	138 (89.0)
Place of work, N (%)	
Stationary department	135 (84.4)
Outpatients’ clinic	25 (15.6)
Nurses’ knowledge about basic human needs, Mdn (IQR)	
General knowledge about people with disabilities	20.0 (3.0)
Physiological needs	20.0 (5.0)
Safety needs	21.0 (3.5)
Affiliative needs	21.0 (5.0)
Self-esteem	21.0 (3.0)
Self-actualization	20.0 (4.0)
Nurses’ overall knowledge about basic human needs	121.0 (15.5)
Nurses’ average knowledge about basic human needs, Mdn (IQR)	4.0 (0.5)

Note: Mdn (IQR) = median (interquartile range); N (%) = absolute number (percentage number). Nurses’ knowledge about basic human needs ranged between five and 25; overall knowledge ranged between 30 and 150; median range was between one and five.

**Table 2:** Loadings of the items of eight factors of the factorial analysis according to the nurses’ knowledge.

	1st	2nd	3rd	4th	5th	6th	7th	8th
3.2. The need for personal hygiene in people with disabilities belongs to safety needs.	0.71							
2.4. Rest and sleep for a person with a disability are essential to relieve tension.	0.70							
3.1. Safety needs are important for survival, but not as physiological needs.	0.64							−0.42
2.2. In general, the physiological needs of people with disabilities need to be satisfied promptly.	0.63				0.47			
2.1. Physiological needs of people with disabilities are divided into survival needs and stimulation needs.	0.61							
2.3. Self-care is one of the important activities in meeting physiological needs for people with disabilities.	0.55							
6.3. In achieving independence and self-actualization of people with disabilities, knowledge about specific problems is essential.		0.78						
6.2. Self-actualization in persons with disabilities includes realizing the best potential that a person has.		0.74						
6.4. In every contact with a person with a disability and their family, we should consider how to increase their level of knowledge.		0.64						
3.3. Meeting the need for personal hygiene affects the development of self-confidence in people with disabilities.		0.58						
6.1. Learning belongs to the need for self-actualization.		0.56	0.51					
1.5. People with disabilities should participate independently and equally in social activities.		0.42		0.46				
1.1. Basic human needs are the same for all people.			0.73					
4.1. It is necessary to respect the spiritual needs of persons with disabilities.			0.66					
4.4. Communication disorders may cause social isolation of people with disabilities.			0.60					
4.5. Verbalization of the feeling of loneliness may indicate the social isolation of a person with a disability.			0.52					
4.2. The need for love and belonging in people with disabilities can be met through communication.			0.42	0.62				
3.5. Avoiding harmful influences and preventing falls, injuries, and infections increases the sense of security in people with disabilities.				0.74				
3.4. Meeting the need for personal hygiene in people with disabilities affects the prevention of infections and pressure ulcers.				0.70				
2.5. The reduced mobility of disabled people is characterized by the need for help from others in movement activities.				0.52				
5.2. Inability to meet basic human needs can have a strong impact on self-esteem, self-confidence, and self-image.					0.71			
5.3 In people whose health damage took place suddenly, there can be a strong decline in self-esteem and self-confidence.					0.60			
5.4. Disturbance of self-concept (i.e., altered self-image) includes a feeling of inferiority.		0.42			0.53			
5.1. * Self-esteem and self-confidence in persons with disabilities are synonymous.						0.79		
1.2. * People with disabilities have different basic human needs when compared with people without disabilities.						0.71		
6.5. * Counseling a person with a disability should be in the sense of suggesting or supervising certain behaviors.	−0.42					0.71		
1.4. People with disabilities have different ways of satisfying their basic human needs.							0.83	
1.3. People with disabilities have different degrees of independence in meeting their needs.							0.80	
5.5. It is necessary to encourage people with disabilities to rely on their strengths.								0.72
4.3. Belonging needs include the religious needs of people with disabilities.			0.52					0.59
Extraction sums of squared loadings (% of variance)	28.2	8.9	7.0	6.1	4.5	4.3	3.9	3.4
Rotation sums of squared loadings (% of variance)	11.4	11.1	9.6	9.5	6.9	6.5	6.0	5.4
Cronbach’s alpha of each of the factors	0.80	0.85	0.73	0.63	0.78	0.68	0.62	0.65

Note: first factor = “knowledge about self-care”, second factor = “knowledge about self-actualization”, third factor = “knowledge about belongingness”, fourth factor = “knowledge about person’ safety”, fifth factor = “knowledge about self-esteem”, sixth factor = “general knowledge and view of advising”, seventh factor = “knowledge about independence”, eighth factor = “knowledge about belief”. * Items (1.2., 5.1., and 6.5.) were reverse-scored. Cronbach’s alpha for the entire questionnaire was α = 0.84.

**Table 3:** Associations between nurses’ sociodemographic characteristics and their knowledge about basic human needs, using linear regression models.

	General Knowledge		Physiological Needs		Safety Needs		Affiliative Needs		Self-Esteem Needs		Self-Actualization	
	β	*p*	β	*p*	β	*p*	β	*p*	β	*p*	β	*p*
Female gender	−0.01	0.912	0.08	0.226	−0.11	0.067	0.05	0.501	−0.01	0.860	0.10	0.121
Age (years)	0.76	0.051	−0.45	0.170	0.16	0.605	−0.17	0.631	0.07	0.852	−0.01	0.976
Length of service (years)	−0.65	0.094	0.40	0.217	−0.08	0.800	0.28	0.431	−0.09	0.792	−0.08	0.812
High-school degree	−0.09	0.686	0.12	0.527	−0.04	0.812	−0.25	0.225	0.29	0.168	−0.44	0.027
Bachelor’s degree	−0.03	0.900	0.07	0.724	−0.11	0.544	−0.19	0.348	0.24	0.228	−0.22	0.250
Stationary department	−0.15	0.071	−0.06	0.362	0.13	0.051	−0.10	0.189	0.13	0.082	−0.06	0.399
University study	0.08	0.310	−0.14	0.028	0.17	0.006	0.03	0.661	0.03	0.691	−0.11	0.117
General knowledge	−	−	0.05	0.545	−0.13	0.080	0.20	0.014	0.15	0.058	0.16	0.036
Physiological needs	0.07	0.545	−	−	0.50	<0.001	0.29	0.002	0.18	0.063	−0.19	0.037
Safety needs	−0.20	0.080	0.54	<0.001	−	−	0.21	0.034	0.12	0.234	0.22	0.026
Affiliative needs	0.25	0.014	0.26	0.002	0.17	0.034	-	-	0.03	0.750	0.14	0.101
Self-esteem	0.19	0.058	0.16	0.063	0.10	0.234	0.03	0.750	-	-	0.41	<0.001
Self-actualization	0.22	0.036	−0.18	0.037	0.19	0.026	0.16	0.101	0.45	<0.001	-	-
Overall model significance	R = 0.57; R^2^ = 0.33; *p* < 0.001		R = 0.73; R^2^ = 0.53; *p* < 0.001		R = 0.75; R^2^ = 0.57; *p* < 0.001		R = 0.68; R^2^ = 0.46; *p* < 0.001		R = 0.67; R^2^ = 0.45; *p* < 0.001		R = 0.71; R^2^ = 0.50; *p* < 0.001	

Note: β = beta coefficient; *p* = *p*-value; R = R value; R^2^ = R-squared.

**Table 4:** Associations between nurses’ sociodemographic characteristics and factorial domains of knowledge about basic human needs, using linear regression models.

	Self-Care		Self-Actualization		Belongingness		Safety		General Knowledge and Advice		Self-Esteem		Independence		Beliefs	
	β	*p*	β	*p*	β	*p*	β	*p*	β	*p*	β	*p*	β	*p*	β	*p*
Female	0.02	0.758	0.07	0.295	0.00	0.975	−0.18	0.012	0.05	0.479	0.03	0.746	−0.07	0.384	0.13	0.073
Age (years)	0.08	0.832	−0.04	0.890	0.37	0.236	−0.66	0.061	0.10	0.790	0.48	0.239	0.05	0.899	0.46	0.204
Length of service (years)	−0.09	0.806	−0.04	0.907	−0.24	0.447	0.69	0.049	−0.16	0.664	−0.51	0.206	0.13	0.759	−0.48	0.181
High-school degree	−0.06	0.779	−0.43	0.017	0.01	0.949	0.26	0.187	0.19	0.344	−0.63	0.005	0.21	0.361	−0.27	0.184
Bachelor’s degree	−0.16	0.431	−0.24	0.181	0.17	0.309	0.16	0.411	0.21	0.294	−0.44	0.045	0.19	0.404	−0.43	0.029
Stationary department	0.07	0.341	−0.01	0.856	−0.11	0.099	0.08	0.270	0.06	0.411	−0.04	0.676	−0.19	0.027	−0.04	0.621
University study	−0.10	0.179	−0.02	0.752	−0.07	0.287	0.10	0.152	−0.02	0.838	−0.12	0.152	0.10	0.246	0.13	0.064
Self-care	-	-	0.14	0.074	0.21	0.004	0.17	0.041	0.04	0.670	−0.30	0.002	0.04	0.657	−0.03	0.700
Self-actualization	0.18	0.074	-	-	0.10	0.243	0.26	0.007	0.39	<0.001	−0.01	0.902	−0.11	0.319	0.19	0.048
Belongingness	0.29	0.004	0.10	0.243	-	-	0.26	0.008	0.20	0.045	0.02	0.834	0.14	0.225	0.35	<0.001
Safety	0.18	0.041	0.21	0.007	0.20	0.008	-	-	0.02	0.857	0.12	0.247	0.15	0.148	0.09	0.328
General knowledge and advice	0.04	0.670	0.31	<0.001	0.15	0.045	0.02	0.857	−	−	−0.01	0.942	0.13	0.181	0.05	0.573
Self-esteem	−0.24	0.002	−0.01	0.902	0.01	0.834	0.09	0.247	−0.01	0.942	−	−	−0.08	0.355	−0.12	0.121
Independent	0.03	0.657	−0.07	0.319	0.08	0.225	0.11	0.148	0.10	0.181	−0.08	0.355	−	−	−0.07	0.346
Beliefs	−0.03	0.700	0.15	0.048	0.27	<0.001	0.08	0.328	0.05	0.573	−0.15	0.121	−0.09	0.346	−	−
Overall model significance	R = 0.61; R^2^ = 0.38; *p* < 0.001		R = 0.71; R^2^ = 0.51; *p* < 0.001		R = 0.73; R^2^ = 0.54; *p* < 0.001		R = 0.64; R^2^ = 0.41; *p* < 0.001		R = 0.47; R^2^ = 0.22; *p* = 0.002		R = 0.61; R^2^ = 0.36; *p* < 0.001		R = 0.44; R^2^ = 0.20; *p* = 0.010		R = 0.62; R^2^ = 0.39; *p* < 0.001	

Note: β = beta coefficient, p = *p*-value; R = R value; R^2^ = R –squared. High-school and bachelor’s degree = master’s degree was the reference group. Stationary department = nurses working in an outpatient’s clinic were the reference group. University study = no study was the reference group. All domains were estimated as the median per factor. Self-care = first factor (domain) grouping nurses’ knowledge. Self-actualization = second factor (domain) grouping nurses’ knowledge. Belongingness = third factor (domain) grouping nurses’ knowledge. Safety = fourth factor (domain) grouping nurses’ knowledge. General knowledge and advice = fifth factor (domain) grouping nurses’ knowledge. Self-esteem = sixth factor (domain) grouping nurses’ knowledge. Independence = seventh factor (domain) grouping nurses’ knowledge. Beliefs = eighth factor (domain) grouping nurses’ knowledge.

## Data Availability

The data presented in this study are available from the corresponding author upon reasonable request.

## Supplementary Materials

The following supporting information can be downloaded at https://www.mdpi.com/article/10.3390/bs13010068/s1: Supplementary Table S1. Knowledge of Basic Human Needs Scale (KBHNS) questionnaire based on Maslow’s hierarchy of basic human needs; Supplementary Table S2. Differences in knowledge about basic human needs according to the nurses’ sociodemographic characteristics (N = 160).
