# Supplementary material for: Are They the Same for All People? Nurses’ Knowledge about the Basic Human Needs of People with Disabilities
Source: Behav Sci (Basel). 2023 Jan 12;13(1):68. doi: 10.3390/bs13010068 (PMC9854844; doi:10.3390/bs13010068)
Supplement: Supplementary file 1 [file behavsci-13-00068-s001.zip › behavsci-2106982-supplementary.pdf]

Supplementary Table S1. Knowledge of Basic Human Needs Scale (KBHNS) questionnaire based on Maslow's hierarchy of basic human needs.

**1. General nurses' knowledge about people with disabilities**
1.1. Basic human needs are the same for all people.
1.2. *People with disabilities have different basic human needs when compared with people without disabilities.
1.3. People with disabilities have different degrees of independence in meeting their needs.
1.4. People with disabilities have different ways of satisfying their basic human needs.
1.5. People with disabilities should participate independently and equally in social activities.
**2. Knowledge about physiological needs of people with disabilities**
2.1. Physiological needs of people with disabilities are divided into survival needs and stimulation needs.
2.2. In general, the physiological needs of people with disabilities need to be satisfied promptly.
2.3. Self-care is one of the important activities in meeting physiological needs for people with disabilities.
2.4. Rest and sleep for a person with a disability are essential to relieve tension.
2.5. The reduced mobility of a disabled person is characterized by the need for help from another person in movement activities.
**3. Knowledge about safety needs of people with disabilities**
3.1. Safety needs are important for survival, but not as physiological needs.
3.2. The need for personal hygiene in people with disabilities belongs to safety needs.
3.3. Meeting the need for personal hygiene affects the development of self-confidence in people with disabilities.
3.4. Meeting the need for personal hygiene in people with disabilities aids in the prevention of infections and pressure ulcers.
3.5. Avoiding harmful influences and preventing falls, injuries, and infections increases the sense of security in people with disabilities.
**4. Knowledge about affiliative needs of people with disabilities**
4.1. It is necessary to respect the spiritual needs of persons with disabilities.
4.2. The need for love and belonging in people with disabilities is met through communication.
4.3. Belonging needs include the religious needs of people with disabilities.
4.4. Communication disorders may cause social isolation of a person with a disability.
4.5. Verbalization of the feeling of loneliness may indicate the social isolation of a person with a disability.
**5. Knowledge about self-esteem needs of people with disabilities**
5.1. *Self-esteem and self-confidence in persons with disabilities are synonymous.
5.2. Inability to meet basic human needs can have a strong impact on self-esteem, self-confidence, and self-image.
5.3. In people whose health damage took place suddenly, there can be a strong decline in self-esteem and self-confidence.
5.4. Disturbance of self-concept (i.e., altered self-image) includes a feeling of inferiority.
5.5. It is necessary to encourage people with disabilities to rely on their strengths.
**6. Knowledge about self-actualization needs of people with disabilities**
6.1. Learning belongs to the need for self-actualization.
6.2. Self-actualization in persons with disabilities includes realizing the best potential that a person has.
6.3. In achieving independence and self-actualization of people with disabilities, knowledge about specific problems is essential.
6.4. In every contact with a person with a disability and their family, we should consider how to increase their level of knowledge.
6.5. *Counseling a person with a disability should be in the sense of suggesting or supervising certain behavior.

Note: The Knowledge of Basic Human Needs Scale (KBHNS) questionnaire assesses nurses' knowledge about the basic human needs of people with disabilities. Answers were scored on a Likert scale ranging from 1 to 5: 1 = completely incorrect; 2 = incorrect; 3 = partially correct; 4 = correct; 5 = completely correct. Items with *(1.2., 5.1.; 6.5.) were scored in reverse. The sum for each of the components ranges from 5 to 25. The sum of all 30 items in the whole questionnaire ranges from 30 to 150. A higher score denotes a higher knowledge about the basic human needs of people with disabilities.

Supplementary Table S2. Differences in knowledge about basic human needs according to the nurses' sociodemographic characteristics (N = 160).

Variable	General knowledge	Physiological needs	Safety needs	Affiliative needs	Self-esteem	Self-actualization
Gender						
Female	20.0 (3.0)	20.0 (5.0)	21.0 (3.5)	21.0 (5.0)	21.0 (3.0)	20.0 (4.0)
Male	18.5 (5.5)	20.0 (6.8)	21.0 (3.3)	21.0 (3.3)	20.0 (1.5)	18.0 (4.5)
*p**	0.630	0.564	0.759	0.532	0.568	0.280
Education						
High school degree	19.0 (4.0)	20.0 (5.0)	21.0 (3.5)	21.0 (4.5)	20.0 (3.0)	20.0 (4.0)
Bachelor's degree	20.5 (4.0)	20.0 (5.0)	21.0 (3.8)	21.0 (5.6)	21.0 (3.01	20.0 (4.0)
Master's degree	21.0 (7.5)	20.5 (9.8)	23.0 (7.0)	23.0 (1.5)	20.0 (5.0)	22.5 (3.8)
p†	0.008	0.331	0.921	0.498	0.267	<0.001
Study at a nursing school						
Yes	19.0 (4.0)	20.0 (6.0)	18.0 (6.0)	20.0 (2.0)	19.0 (3.0)	20.0 (4.0)
No	20.0 (3.0)	20.0 (5.0)	21.0 (3.0)	21.0 (5.0)	20.1 (3.0)	20.0 (3.5)
*p**	0.395	0.614	0.003	0.051	0.112	0.873
Place of work						
Stationary department	20.0 (3.0)	20.0 (5.0)	21.0 (4.0)	21.0 (5.0)	20.0 (3.0)	20.0 (4.0)
Outpatients' clinic	19.0 (5.0)	20.0 (4.0)	21.0 (5.0)	22.0 (3.5)	21.0 (2.5)	19.0 (4.5)
*p**	0.152	0.820	0.059	0.668	0.309	0.852
Length of service (years)						
Fewer than 10	19.0 (4.0)	19.0 (3.5)	20.0 (3.0)	21.0 (3.5)	20.0 (3.5)	20.0 (4.0)
11-20	20.0 (3.5)	20.0 (6.0)	21.0 (4.0)	21.0 (6.0)	21.0 (5.0)	21.0 (5.0)
21-30	21.0 (2.5)	21.0 (6.0)	21.0 (4.5)	23.0 (5.0)	21.0 (2.0)	20.0 (3.0)
31 and over	19.0 (3.5)	20.0 (4.5)	21.0 (4.0)	21.0 (4.5)	21.0 (3.0)	20.0 (3.0)
p†	0.267	0.347	0.015	0.194	0.590	0.550

Note: The parameter estimates in each column are the median (interquartile range) of nurses' knowledge; $p < 0.005$; *Mann–Whitney U test; †Kruskal–Wallis test.
